# Inactivation and sub-lethal injury of *salmonella *typhi, *salmonella *typhimurium and *vibrio cholerae *in copper water storage vessels

**DOI:** 10.1186/1471-2334-11-204

**Published:** 2011-07-27

**Authors:** Riti Sharan, Sanjay Chhibber, Robert H Reed

**Affiliations:** 1 Centre for Plant and Water Science, Faculty of Sciences, Engineering and Health, CQUniversity, Rockhampton, Queensland 4702, Australia; 2 Department of Microbiology, Panjab University, Sector-14, Chandigarh-160014, India

## Abstract

**Background:**

This study provides information on the antibacterial effect of copper against the water-borne pathogens *Salmonella *Typhi, *Salmonella *Typhimurium and *Vibrio cholerae*.

**Methods:**

Suspensions of each pathogen were kept in water within a traditional copper vessel at 30°C for 24 h. Samples were withdrawn, diluted and plated onto suitable growth media. Conventional enumeration of healthy (uninjured) bacteria was carried out using standard aerobic incubation conditions. Additionally, reactive oxygen species-neutralised (ROS-n) conditions were achieved by adding the peroxide scavenger sodium pyruvate to the medium with anaerobic incubation, to enumerate uninjured (ROS-insensitive) and injured (ROS-sensitive) bacteria. Differences between log-transformed means of conventional (aerobic) and ROS-n counts were statistically evaluated using *t *tests.

**Results:**

Overall, all three pathogens were inactivated by storage in copper vessels for 24 h. However, for shorter-term incubation (4-12 h), higher counts were observed under ROS-n conditions than under aerobic conditions, which demonstrate the presence of substantial numbers of sub-lethally injured cells prior to their complete inactivation.

**Conclusions:**

The present study has for the first time confirmed that these bacterial pathogens are inactivated by storage in a copper vessel within 24 h. However, it has also demonstrated that it is necessary to account for short-term sub-lethal injury, manifest as ROS-sensitivity, in order to more fully understand the process. This has important practical implications in terms of the time required to store water within a copper vessel to completely inactivate these bacteria and thereby remove the risk of water-borne disease transmission by this route.

## Background

According to the World Health Organization, an estimated 4.1% of the total global burden of disease is contributed by diarrhoeal illness: around 88% of that burden is due to unsafe water supply, sanitation and hygiene, with children in developing countries being the most common victims [1]. Though there has been improvement in providing safe drinking water in developing nations such as India, the adverse impact of unsafe water continues. Today, inadequate access to clean drinking water and sanitation are among the biggest environmental problems in India, threatening both urban and rural populations; children suffering from microbial contamination of drinking water supplies are exposed to a range of viral, bacterial and protozoal pathogens [2-4].

Among the water-borne pathogenic bacteria prevalent in developing nations, *Salmonella enterica *is of considerable significance, with approximately 22 million cases of enteric fever caused by *Salmonella *Typhi in 2002 [5] and approximately 5.5 million cases of enteric fever caused each year by *Salmonella *Paratyphi A, B or C [6]. In a study conducted on urban drinking water supply systems in Nepal, *Salmonella *was detected in 42 out of 300 water samples, with the predominant serotype being *Salmonella *Typhimurium [7]. Typhoid fever is one of the main public health problems in urban slums in India [5]. In a 2006 study in Kolkata, it was observed that typhoid fever mainly occurred in younger men, and that the absence of hygienic habits such as hand washing was a major route of transmission [8]. Similarly, cholera outbreaks have been reported from rural areas in India with unhygienic environmental conditions, inadequate sanitation and unsafe water supplies [9] and toxigenic strains of *Vibrio cholerae *O1 has been responsible for many large water-borne outbreaks of cholera on a global scale [10].

Ancient scriptures dating back to around 2000 BC highlight various practices of water treatment by filtration, exposure to sunlight and storage in brass and copper vessels [11]. Consequently, the antibacterial potential of copper has been exploited since these ancient times. In contrast to the low sensitivity of human tissue to copper, microorganisms generally are extremely susceptible to copper, making it suitable for water disinfection [12]. Previous studies have investigated the inactivation and injury of faecal indicator bacteria such as *Escherichia coli *and *Enterococcus faecalis *during short-term storage in brass vessels [11,13], with reactive oxygen species-neutralized (ROS-n) conditions consistently giving higher counts than aerobic counts indicating that conventional plate counting is not sufficient to enumerate sub-lethally injured bacteria. A study from south India [14] also described decreased bacterial contamination of water stored in a brass container, though without investigating the potential effects of sub-lethal injury. More recent research has reported the inactivation of *E. coli, S*. Typhi and *V. cholerae *after overnight storage in a copper vessel [15] though, again, the possibility of sub-lethal injury and lack of growth of injured cells under conventional enumeration conditions was not considered.

There are several reports of *V. cholerae *entering a dormant or a viable-but non-culturable (VNC) state due to nutrient deprivation and/or stressful environmental conditions [16,17]. However, an alternative explanation for the VNC state involves sub-lethal injury, as demonstrated for *Salmonella *Typhimurium following thermal and non-thermal food preservation procedures, where sub-lethal injury was demonstrated by plating on different selective media such as trypticase soy agar and violet red bile glucose agar, with the latter giving lower counts [18]. Conventional microbiological enumeration practices using aerobic culture does not take into account sub-lethal injury of the bacteria, which can be demonstrated through ROS-neutralisation techniques.

The present study was designed to investigate not only the dynamics of inactivation of *S*. Typhi, *S*. Typhimurium and *V. cholerae *in water stored in copper vessels but also to quantify the extent of any sub-lethal injury caused to these bacteria during storage in the vessels. Such sub-lethal injury is manifest in terms of ROS-sensitivity and counteracted using ROS-n enumeration conditions [19]. The findings of the present study have important implications in relation to the practical use of copper vessels in rural areas of India and elsewhere, in terms of the time required to completely inactivate both healthy and injured bacteria.

## Methods

### Bacterial strains and culture preparation

*S*. Typhimurium MTCC1251 and MTCC98, *S*. Typhi MTCC733 and *V. cholerae *MTCC3906 were obtained from Institute of Microbial Technology, Chandigarh while *S*. Typhi Ty2 and *V. cholerae *O1 were obtained from the Central Research Institute, Kasauli. All strains were studied at Panjab University. Stocks were maintained by sub-culturing every 15-20 days on nutrient agar (HiMedia, Mumbai, India). For experimental procedures, 1 CFU 100 ml^-1 ^NB was incubated for 18 h at 37°C without shaking. In this way, the cells reached stationary phase in an essentially anaerobic environment [19]. This approach enables the cells to grow by fermentative metabolism, in contrast to aerobic shake cultures, which would grow by respiratory metabolism. Using cells grown under fermentative conditions enables the effects of oxygen sensitivity to be studied in the experiments, since such cells are likely to show heightened responses to oxygen. The pH of the nutrient broth was adjusted to be slightly alkaline (pH 7.5) using 0.01 mol L^-1 ^HEPES/NaOH [4-(-2 Hydroxyethyl)-1-piperazine ethanesulfonic acid, HiMedia, Mumbai, India] for *V. cholerae*. The culture was then centrifuged at 5300 × *g *for 5 min at 5°C and rinsed twice with 0.85% NaCl to remove all traces of growth medium. The pellet was then suspended in the same volume of sterile distilled water and then diluted to 1:100 for experimental purposes, i.e 100 ml of bacterial suspension was added to 10 litres of sterile distilled water at pH 7.0 for *S*. Typhi, *S*. Typhimurium and 7.5 for *V. cholerae *to give a to give a final density of 10^6 ^-10^7 ^colony forming units per millilitre (CFU mL^-1^) at 0 h.

### Water source and Storage vessel

Sterile distilled water was used for all experimental procedures. This water was adjusted to pH 7.0 for *S*. Typhi and *S*. Typhimurium and to pH 7.5 for *V. cholerae*. Copper vessels (12 litre capacity), obtained from the local market in Chandigarh were disinfected, scrubbed and rinsed thoroughly to remove any adherent contamination from the inner surface. In order to mimic the rural setting in a laboratory by keeping the cleansing and disinfecting step simple, easy and cost-effective, the present method of rinsing with water, disinfecting and rinsing thoroughly three times with several litres of sterile distilled water each time before use was carried out [20]. Chemical analysis of the composition of the copper vessels was carried out using the Test-Master Pro, WAS Worldwide Analytical Systems AG, Wellesweg, Germany and was found to contain 95% copper and 4.59% zinc. The mouths of all vessels were kept covered with sterile paper during the test procedure, to prevent airborne contamination of the water stored within. No contaminant colonies were seen during experimentation, confirming the effectiveness of this approach.

### Laboratory experiments

Suspensions of each bacterial strain were stored in water in copper vessels at 30°C for 24 h. Samples were withdrawn at 0, 2, 4, 6, 8, 10, 12 and 24 h, and serial decimal dilutions were prepared for each sample, to cover the dilution range 10^0^-10^-4^. Triplicate nutrient agar plates with and without 0.05% w/v sodium pyruvate, peroxide quencher [19,21], were prepared for each sample/dilution using a modified Miles and Misra surface droplet method [11], where 20 μl of each dilution was placed onto the surface of pre-dried agar medium within a petri plate using a calibrated micropipette. The droplets were then spread across the surface of the medium using an inoculation loop, with 3 droplets per plate. Each drop is of 20 μl volume, making it 60 μl when the results for triplicate drops are added together. Here the loop was simply used to spread out the whole droplet, as an alternative to a 'spreader' (due to the smaller size of the droplet). Suspensions of each bacterial strain were stored in glass vessels under identical conditions and were processed similarly for enumeration. Each experiment was repeated three times with each strain.

### Enumeration

Nutrient agar plates without 0.05% w/v sodium pyruvate were incubated in a conventional aerobic incubator at 37°C for 24 h while nutrient agar plates supplemented with 0.05% w/v sodium pyruvate were incubated anaerobically in an anaerobic jar (HiMedia System Mark II) at 37°C for 24 h (ROS-n conditions) followed by further 24 h incubation at 37°C under normal aerobic conditions to ensure that all colonies were large enough to count. A non-selective medium was able to be used as the experiments were carried out with a pure suspension of a single bacterium [no contaminants were present]. Colonies were counted following the appropriate incubation period and the data expressed as CFU ml^-1 ^by correcting for dilution and volume. The minimum count (detection limit) in all the experiments was 1 colony forming unit (CFU) in 3 × 0.02 ml aliquots (0.06 ml) which corresponds to16.7 CFU mL^-1 ^(colony forming unit = {C/V} × M) or 1.222 when expressed as a log value. This has been represented in all the graphs as a dotted horizontal trend line. At any given time point during the course of the experiment, if the counts were below 1 CFU per 0.06 ml, they were regarded as counts below the minimum detection limit (< 1.222). It is important to appreciate that such values cannot be shown directly on a log scale: in experiments where the counts fell below the detection limit, the plotted curve appears to stop at the last recorded positive count but it is important to realise that this does not mean that samples were not taken and processed for time point beyond this value, but that the values cannot be shown, since they are < 1.222.

### Statistical analysis

Colony counts are expressed as mean log_10_CFU ml^-1 ^with 95% confidence limits. 95% confidence limits were used for error bars in the graphs. Differences between log-transformed means of conventional (aerobic) and ROS-n counts were statistically evaluated using *t *tests. The *t *and *p *value for two sets of log values at the same time point was calculated using the *t test: paired two sample for means *function in Microsoft Excel spreadsheet.

## Results

**Figure 1:**
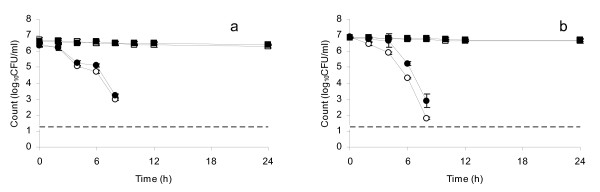
**Plate counts of (a) *S*. Typhi 733 and (b) *S*. Typhi Ty2 in distilled water at pH 7.0, 30°C stored in a copper vessel and enumerated under aerobic conditions (open circles) and ROS-n conditions (closed circles); (a) *S*. Typhi 733 and (b) *S*. Typhi Ty2 in distilled water at pH 7.0, 30°C stored in a glass vessel and enumerated under aerobic conditions (open squares) and ROS-n conditions (closed squares) Error bars represent 95% confidence limits (*n *= 3)**. No counts were obtained at 24 h under any conditions. No counts were obtained after 8 h for bacteria kept in water in the copper vessel.

Figure 1a and 1b show the inactivation and sub-lethal injury of *S*. Typhi 733 and *S*. Typhi Ty2 in water stored in a copper vessel for up to 24 h, enumerated under normal aerobic conditions and ROS-n conditions. Counts for both strains fell below the minimum detection limit by 10 h with the degree of sub-lethal injury, demonstrated in terms of the difference between aerobic and ROS-n counts, being more pronounced for *S*. Typhi Ty2 at 48 h. This difference was statistically significant at 4 h (*t *= 8.21, *p *= 0.01) and beyond. A statistically significant difference between aerobic and ROS-n counts of *S*. Typhi 733 was also observed at 4 h (*t *= 5.26, *p *= 0.03). No counts were detected for both strains after 8 h under aerobic conditions and ROS-n conditions.

**Figure 2:**
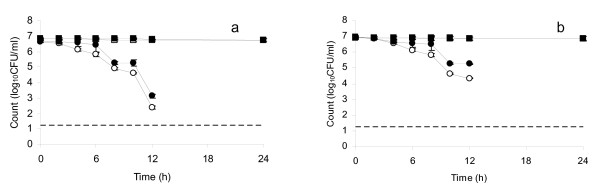
**Plate counts of (a) *S*. Typhimurium 98 and (b) *S*. Typhimurium 1251 in distilled water at pH 7.0, 30°C stored in a copper vessel and enumerated under aerobic conditions (open circles) and ROS-n conditions (closed circles); (a) *S*. Typhimurium 98 and (b) *S*. Typhimurium 1251 in distilled water at pH 7.0, 30°C stored in a glass vessel and enumerated under aerobic conditions (open squares) and ROS-n conditions (closed squares)**. Error bars represent 95% confidence limits (*n *= 3). No counts were obtained at 24 h under any conditions. No counts were obtained after 12 h for bacteria kept in water in the copper vessel.

*S*. Typhimurium 98 and *S*. Typhimurium 1251 (Figure 2a and 2b) were less rapidly inactivated than the strains of *S*. Typhi, with counts at 12 h but not at 24 h. A significant difference was observed between aerobic and ROS-n counts at 12 h (*t *= 15.55, *p *= 0.004 for *S*. Typhimurium 98 and *t *= 23.93, *p *= 0.002 for *S*. Typhimurium 1251), indicating sub-lethal injury to both strains. Counts for both strains of *S*. Typhimurium fell below the minimum detection limit after 12 h under aerobic and ROS-n conditions.

**Figure 3:**
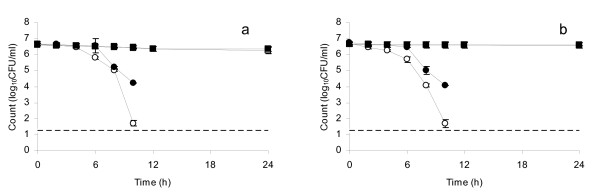
**Plate counts of (a) *V. cholerae *3906 and (b) *V. cholerae *O1 in distilled water at pH 7.5, 30°C stored in a copper vessel and enumerated under aerobic conditions (open circles) and ROS-n conditions (closed circles); (a) *V. cholerae *3906 and (b) *V. cholerae *O1 in distilled water at pH 7.5, 30°C stored in a glass vessel and enumerated under aerobic conditions (open squares) and ROS-n conditions (closed squares)**. Error bars represent 95% confidence limits (*n *= 3). No counts were obtained at 24 h under any conditions. No counts were obtained after 10 h for bacteria kept in water in the copper vessel.

For *V. cholerae *3906 and *V. cholerae *O1 there was minimal change in counts over the first 4 h of storage, followed by a more rapid decrease with both aerobic and ROS-n counts falling below minimum detection limit after 10 h (Figure 3a and 3b). Compared to *S*. Typhi and *S*. Typhimurium, the two test strains of *V. cholerae *demonstrated the largest difference between aerobic and ROS-n counts at 10 h (*t *= 60.48, *p *= 0.0003 for *V. cholerae *3906 and *t *= 4.0.20, *p *= 0.0006 for *V. cholerae *O1). Such differences represent a substantial number of sub-lethally injured cells, e.g. at 10 h the counts were 5 × 10^2 ^CFU ml^-1 ^for *V. cholerae *3906 under aerobic conditions and 1.5 × 10^4 ^CFU ml^-1 ^under ROS-n conditions, with equivalent counts for *V. cholerae *O1 of 5 × 10^2 ^CFU ml^-1 ^and 1.0 × 10^4 ^CFU ml^-1^.

Overall, all test strains were completely inactivated (with no counts obtained under aerobic or ROS-n conditions) by storage in water kept in a copper vessel for 24 h, though all demonstrated sub-lethal injury (sensitivity to aerobic enumeration conditions) during shorter-term exposure. In contrast, storage of the same strains in water maintained in glass vessels resulted in no substantial change in aerobic or ROS-n counts up to 24 h (control data shown in Figures 1, 2, 3).

## Discussion

Despite global initiatives in water and sanitation, enteric fever and cholera remain major public health problems in many developing countries [22,23], primarily due to lack of adequate sanitation facilities and safe drinking water, alongside issues related to personal hygiene [24]. Not only are some sources of drinking water contaminated, the likelihood of post-collection contamination during transport and/or storage at the household level is also high [25]. Given the large population of developing nations such as India and the scarcity of water resources, there is an unmet need to address the issue of point-of-use disinfection of drinking water, especially in rural settings. One of the proposed solutions to this problem was installation of boreholes and community wells. However, these protected sources of water are usually located far away from homes and require storage for some time before consumption [26], leaving the problem of post-collection contamination.

The present study has established the antibacterial effect of copper against some of the most important water-borne pathogens of developing nations, namely *S*. Typhi, *S*. Typhimurium and *V. cholerae*. We have also demonstrated that injury is caused to the bacteria during short-term storage, and that this must be taken into account in order to obtain a comprehensive view of the antibacterial process, since ROS-sensitive bacteria are not detected by conventional procedures, and would therefore be missed during counting using standard laboratory methods and media. This is a significant finding as the sub-lethally injured microorganisms maybe as important as their healthy counterparts as they could have the potential to resuscitate in a favourable environment [27]. The frequency of isolation of *V. cholerae *and *V. vulnificus *is affected by environmental conditions [28] and it has been proposed that such organisms may enter a viable-but-non-culturable (VNC) state [29]. However, such an interpretation is typically based on conventional aerobic counts, and the ROS sensitivity of sub-lethally injured cells of *V. vulnificus *[30] provides an alternative possibility. Thus the resuscitation of starved *V. vulnificus *[31] and *V. parahaemolyticus *[32] through supplementation of the growth medium with pyruvate- and/or catalase reinforces the need to consider ROS-n conditions for bacterial enumeration.

The findings of the present study are significant at the practical level, since they provide information that extends the findings of earlier studies, based on conventional aerobic counts [14,15]. While there is a possibility that cells of pathogenic bacteria may not be completely inactivated when water is stored for short-term periods in copper vessels, our results clearly demonstrate that 24 h is sufficient to reduce counts of all tested strains of *S*. Typhi, *S*. Typhimurium and *V. cholerae *by > 10^5 ^CFU ml^-1^, dropping below the minimum detection limit by this time. Consequently, 24 h storage is recommended in preference to shorter-term storage, e.g. overnight. Our previous studies have demonstrated leaching of copper ions when water is stored at 35°C at pH 7.0. The total dissolved copper was measured using a Perkin-Elmer Analyst atomic absorption spectrophotometer, Perkin-Elmer, USA at the Post Graduate Institute of Medical Sciences, Chandigarh [33]. Clearly, the inactivation is not due to nutritional stress, as in a glass vessel there is no lethality. The levels of copper in the vessel are not sufficient to be of concern in any nutritional or osmoric sense - they are still low, but enough to injure and then inactivate the bacteria.

## Conclusions

Effective point-of-use water disinfection methods are required to reduce the incidence of diarrhoeal diseases in developing nations such as India. We have demonstrated the value of using traditional copper storage vessels to inactivate the water-borne pathogens responsible for typhoid fever and cholera, while also highlighting the occurrence of sub-lethal injury during storage for periods of less than 24 h. The application of improved methods for water storage at the household level are likely to have a significant impact on the overall health of the community [25,26].

## Conflicts of Interest

The authors declare that they have no competing interests.

## Authors' contributions

RR and SC conceived the research topic. RS designed and conducted the laboratory experiments and interpreted the results. Data analysis was carried out by RR and RS. All authors co-drafted the manuscript and approved the final text. RS is guarantor of the paper.

## Pre-publication history

The pre-publication history for this paper can be accessed here:

http://www.biomedcentral.com/1471-2334/11/204/prepub

## References

[B1] World Health Organization (WHO) Burden of disease and cost-effectiveness estimates 2004 http://www.who.int/water_sanitation_health/diseases/burden/en/index.html

[B2] Steinberg EB Mendoza CE Glass R Arana B Lopez MB Mejia M Gold BD Priest JW Bibb W Monroe SS Bern C Bell BP Hoekstra RM Klein R Mintz ED Luby S Prevalence of infection with waterborne pathogens: A seroepidemiologic study in children 6-36 months old in San Juan Sacatepequez, Guatemala Am J Trop Med Hyg 2004 70 83 88 14971703

[B3] Nwachcuku N Gerba CP Emerging water-borne pathogens: can we kill them all? Curr Opinion Biotech 2004 15 175 180 10.1016/j.copbio.2004.04.010 PMC7134665 15193323

[B4] Faundez G Troncoso M Navarrete P Figueroa G Antimicrobial activity of copper surfaces against suspensions of *Salmonella enterica *and *Campylobacter jejuni* BMC Microbiol 2004 4 19 10.1186/1471-2180-4-19 15119960 PMC411034

[B5] Crump JA Okoth GO Slutsker L Ogaja DO Keswick BH Luby SP Effect of point-of-use disinfection, flocculation and combined flocculation-disinfection on drinking water quality in western Kenya J Appl Microbiol 2004 97 225 231 10.1111/j.1365-2672.2004.02309.x 15186460

[B6] Maskey AP Day JN Tuan PQ Thwaites GE Campbell JI Zimmerman M Farrar JJ Basnyat B *Salmonella enterica *serovar Paratyphi A and *S. enterica *serovar Typhi cause indistinguishable clinical syndromes in Kathmandu, Nepal Clin Infect Dis 2006 42 1247 1253 10.1086/503033 16586383

[B7] Bhatta DR Bangtrakulnonth A Tishyadhigama P Saroj SD Bandekar JR Hendriksen RS Serotyping, PCR, phage-typing and antibiotic sensitivity testing of *Salmonella *serovars isolated from urban drinking water supply systems of Nepal Lett Appl Microbiol 2007 44 588 594 10.1111/j.1472-765X.2007.02133.x 17576218

[B8] Mandal S Mandal MD Pal NK Antimicrobial resistance pattern of *Salmonella typhi *isolates Kolkata, India during 1991-2001: a retrospective study Jap J Infect Dis 2002 55 58 59 12082312

[B9] Sur D Seidlein L Manna B Dutta S Deb AK Sarkar BL Kanungo S Deen JL Ali M Kim DR Gupta VK Ochiai RL Tsuzuki A Acosta CJ Clemens JD Bhattacharya SK The malaria and typhoid fever burden in the slums of Kolkata, India: data for a prospective community-based study Tran R Soc Trop Med Hyg 2006 100 725 733 10.1016/j.trstmh.2005.10.019 16455118

[B10] Alam M Sultana M Nair GB Siddique AK Hasan NA Sack RB Sack DA Ahmed KU Sadique A Watanabe H Grim CJ Huq A Colwell RR Viable but nonculturable *Vibrio cholerae *O1 in biofilms in the aquatic environment and their role in cholera transmission PNAS, USA 2007 104 17801 17806 10.1073/pnas.0705599104 PMC2077051 17968017

[B11] Tandon P Chhibber S Reed RH Survival and detection of the faecal indicator bacterium *Enterococcus faecalis *in water stored in traditional vessel Indian J Med Research 2007 125 557 566 17598942

[B12] Pyle BH Broadaway SC McFeters GA Efficacy of copper and silver ions with iodine in the inactivation of *Pseudomonas cepacia* J Appl Bacteriol 1992 72 71 79 1371772 10.1111/j.1365-2672.1992.tb04884.x

[B13] Tandon P Chhibber S Reed RH Inactivation of *Escherichia coli *and coliform bacteria in traditional brass and earthernware water storage vessels Ant van Leeuw 2005 88 35 48 10.1007/s10482-004-7366-6 15928975

[B14] Brick T Primrose B Chandrasekhar R Roy S Muliyil J Kang G Water contamination in urban south India: household storage practices and their implications for water safety and enteric infections Int J Hyg Environ Health 2004 207 473 480 10.1078/1438-4639-00318 15575563

[B15] Sudha VBP Singh KO Prasad SR Venkatasubramanian P Killing of enteric bacteria in drinking water by a copper device for use in home: laboratory evidence Trans R Soc Trop Med Hyg 2009 103 819 822 10.1016/j.trstmh.2009.01.019 19230946

[B16] Wong H Wang P Chen SY Chiu SW Resuscitation of viable but non-culturable *Vibrio parahaemolyticus *in a minimum salt medium FEMS Microbiol Letters 2004 233 269 275 10.1111/j.1574-6968.2004.tb09491.x 15063495

[B17] Oliver JD Fratamico PM, Bhunia AK Viable but nonculturable bacteria in food environments Food Borne Pathogens: Microbiology and Molecular Biology 2005 Horizon Scientific Press, Norfolk, U.K.

[B18] Wuytack EY Phuong LDT Aertsen A Reyns KMF Marquenie D De Ketelaere B Masschalck B Van Opstal I Diels AMJ Michiels CW Comparison of sub-lethal injury induced in *Salmonella enterica *serovar Typhimurium by heat and by different nonthermal treatments J Food Protection 2003 66 31 37 10.4315/0362-028x-66.1.31 12540178

[B19] Khaengraeng R Reed RH Oxygen and photoinactivation of *Escherichia coli *in UVA and sunlight J Appl Microbiol 2005 99 39 50 10.1111/j.1365-2672.2005.02606.x 15960663

[B20] Tandon P Chhibber S Reed RH Survival and detection of the faecal indicator bacterium *Enterococcus faecalis *in water stored in traditional vessel Indian J Med Res 2007 125 557 566 17598942

[B21] Stephens PJ Druggan P Caron NV Stressed *Salmonella *are exposed to reactive oxygen species from two independent sources during recovery in conventional culture media Int J Food Microbiol 2000 60 269 285 10.1016/S0168-1605(00)00345-7 11016616

[B22] Ochiai RL Wang XY von Seidlein L Yang J Bhutta ZA Bhattacharya SK Agtini M Deen JL Wain J Kim DR Ali M Acosta CJ Jodar L Clemens JD *Salmonella *Paratyphi A rates, Asia Emerg Infect Dis 2005 11 1764 1766 16318734 10.3201/eid1111.050168 PMC3367370

[B23] Gaffga NH Tauxe RV Mintz ED Cholera: A new homeland in Africa? Am J Trop Med Hyg 2007 77 705 713 17978075

[B24] Thompson T Sobsey M Bartram J Providing clean water, keeping water clean: an integrated approach Int J Env Health Res 2003 13 S89 S94 10.1080/0960312031000102840 12775384

[B25] Trevett AF Carter RC Targeting appropriate interventions to minimize deterioration of drinking-water quality in developing countries J Health Popul Nutr 2008 26 125 138 18686547 PMC2740669

[B26] Eshcol J Mahapatra P Keshapagu S Is fecal contamination of drinking water after collection associated with household water handling and hygiene practices? A study of urban slum households in Hyderabad, India J Water Health 2009 7 145 154 10.2166/wh.2009.094 18957783

[B27] Wu J Doan H Cuenca MA Investigation of gaseous ozone as an anti-fungal fumigant for stored wheat J Chem Tech & Biotechnol 2006 81 1288 1293 10.1002/jctb.155021727015

[B28] Constantin de Magny G Long W Brown CW Hood RR Huq A Murtugudde R Colwell R Predicting the distribution of *Vibrio *spp. In the Chesapeake Bay: A *Vibrio cholerae *case study EcoHealth 2009 6 378 89 10.1007/s10393-009-0273-6 20145974 PMC2880626

[B29] Chaiyanan S Chaiyanan S Huq A Maguel T Colwell RR Viability of the nonculturable *Vibrio cholerae *O1 and O139 Syst App Microbiol 2001 24 331 341 10.1078/0723-2020-00032 11822667

[B30] Bogosian G Aardema ND Bourneuf EV Morris PJL O'Neil JP Recovery of hydrogen peroxide-sensitive culturable cells of *Vibrio vulnificus *gives the appearance of resuscitation from a viable but nonculturable state J Bacteriol 2000 182 5070 5075 10.1128/JB.182.18.5070-5075.2000 10960089 PMC94653

[B31] Bang W Drake MA Jaykus LA Recovery and detection of *Vibrio vulnificus *during cold storage Food Microbiol 2007 24 664 670 10.1016/j.fm.2006.12.002 17418319

[B32] Mizunoe Y Wai SN Ishikawa T Takade A Yoshida S Resuscitation of viable but nonculturable cells of *Vibrio parahemolyticus *induced at low temperature under starvation FEMS Microbiol Letters 2000 186 115 120 10.1111/j.1574-6968.2000.tb09091.x 10779722

[B33] Sharan R Chhibber S Attri S Reed RH Inactivation and injury of *Escherichia coli *in a copper water storage vessel: effects of temperature and pH Ant van Leeuwen 2010 97 91 97 10.1007/s10482-009-9395-7 19924559

